# Association between Oral Health and Depressive Symptoms in Chinese Older Adults: The Mediating Role of Dietary Diversity

**DOI:** 10.3390/nu16081231

**Published:** 2024-04-21

**Authors:** Jiaxu Lou, Jian Wang, Yingjie Fu, Derong Huang, Mei Liu, Ruonan Zhao, Jiahui Deng

**Affiliations:** 1 Centre for Health Management and Policy Research, School of Public Health, Cheeloo College of Medicine, Shandong University, Jinan 250012, China; 202236544@mail.sdu.edu.cn (J.L.); yingjiefu@mail.sdu.edu.cn (Y.F.); huangdr@mail.sdu.edu.cn (D.H.); 202216572@mail.sdu.edu.cn (M.L.); 202236561@mail.sdu.edu.cn (R.Z.); 202236537@mail.sdu.edu.cn (J.D.); 2 NHC Key Lab of Health Economics and Policy Research, Shandong University, Jinan 250012, China

**Keywords:** depressive symptoms, denture, toothbrushing, dietary diversity, older adults

## Abstract

Diet is a modifiable factor in healthy population aging. Additionally, oral health and diet are important factors affecting depressive symptoms. To assess the mediating role of dietary diversity (DD) in oral health and depressive symptoms in older adults, we selected 8442 participants aged ≥ 65 years from the 2018 Chinese Longitudinal Health Longevity Survey (CLHLS) for a cross-sectional study. Depressive symptoms were determined based on scores on the 10-item Center for Epidemiologic Studies Depression Scale (CESD-10). Dietary diversity scores (DDS) were established based on the frequency of intake of food groups. Oral health was measured by denture use and toothbrushing frequency. Stepwise multiple linear regression and PROCESS macros were used for mediated effects analysis and testing. The sample had a positive detection rate of 44.1% for depressive symptoms, 40.8% for denture use, and 41.9% for once-a-day toothbrushing. Denture use (ρ = −0.077, *p* < 0.01) and toothbrushing frequency (ρ = −0.115, *p* < 0.01) were negative predictors of depressive symptoms in older adults. DD significantly mediated the association between denture use (indirect effect −0.047; 95%CI: −0.068–0.028; *p* < 0.001), toothbrushing frequency (indirect effect −0.041; 95%CI: −0.054–0.030; *p* < 0.001), and depressive symptoms. Denture use and toothbrushing frequency not only directly reduce the risk of depressive symptoms in older adults, but also indirectly affect depressive symptoms through DD.

## 1. Introduction

With the global increase in life expectancy, the unprecedented acceleration of the population aging process has become an important social issue in China. According to the World Health Organization (WHO), by the middle of the twenty-first century, the population aged 65 and over is expected to account for 16.7% of the world’s total population [1]. The older population is highly vulnerable to illness or other negative life events. They suffer from more complex and severe psychological trauma [2]. Depressive symptoms such as persistent depression, sleep problems, reduced social activities, and even suicide are common mental health conditions for them [3]. In China, previous regional surveys have shown a prevalence of 40% approximately [4]. Older adults are facing more serious mental health problems. Due to the limited effectiveness of medication for depressive symptoms, a growing number of studies have focused on modifiable risk factors for depressive symptoms.

In previous studies on the factors influencing depressive symptoms in older adults, chronic diseases are important risk factors [5]. Among chronic diseases, oral diseases are a major public health problem affecting more than 3.5 billion people worldwide [6]. With the progression of aging, the oral structure and function of the older adults have deteriorated. As a result, tooth loss is very common in older adults with a global prevalence of 23.7% [7]. Oral disease can lead to oral dysfunction, resulting in discomfort, pressure, nutritional deficiencies and poor quality of life [8,9,10], and psychological atrophy and reduced social interaction in older adults [11], which effectively affects the occurrence of depressive symptoms in the older adults [6]. Despite its critical relevance to people’s quality of life and well-being, the prevention and management of oral diseases has long been under-emphasized in the overall health agenda [12]. This neglect of oral health needs to be urgently changed.

Diet is a modifiable factor in healthy population aging, especially in the older adults. Dietary diversity (DD) is recognized as an important determinant of health and survival. Not only that, but most dietary guidelines around the world recommend increasing DD [13,14,15]. DD refers to the consumption of different food group quantities over a given period of time, and it is assessed using the Dietary Diversity Score (DDS), which is a concise and efficient indicator of dietary quality [16]. Poor oral health in older adults will lead to an increase in the variety of unchewable foods [17], which affects the balanced absorption of nutrients [17,18,19]. Several studies have shown a negative association between DDS and depressive symptoms in older adults [20,21,22], and monotonous and unhealthy dietary patterns are directly associated with an increased risk of developing depressive symptoms [20]. At present, the mechanism of oral health on the depressive symptoms in older adults is not clear, and research on the mediating role of diet is still lacking. Therefore, exploration of the relationship is necessary. Indeed, even small improvements in diet can make a big difference in the mental health and well-being of older adults. A comprehensive understanding of oral health, diet, and their links to depressive symptoms can help to develop evidence-based recommendations for older adults to promote mental health and improve quality of life. In order to examine whether the emotional beneficial effects of oral health and DD still apply to older adults, the following research hypothesis is proposed based on existing studies.

H1: Denture use negatively predicted depressive symptoms in older adults. H2: Toothbrushing frequency negatively predicted depressive symptoms in older adults. H3: DD mediated the relationship between denture use and depressive symptoms in older adults. H4: DD mediated the relationship between toothbrushing frequency and depressive symptoms in older adults. Therefore, this study aims to explore the effects of oral health on depressive symptoms in Chinese older adults in two pathways. The first is whether denture use affects depressive symptoms in older adults through DD. The second is whether toothbrushing frequency affects depressive symptoms in older adults through DD.

## 2. Methods

### 2.1. Data Sources and Sample

A cross-sectional study was conducted based on the seventh wave of the Chinese Longitudinal Health Longevity Survey (CLHLS) in 2018. The CLHLS was organized by the Center for Healthy Aging and Development Research/National Development Research Institute, Peking University, using a multistage whole-group random sample for a nationwide survey of factors of longevity in old age among Chinese aged 65 years and older, and is the earliest started and longest continued (1998–2018) social science survey. The questionnaire data were collected in the form of household visits. Investigators obtained basic health status by interviewing older adults and their closest relatives or caregivers. In this way, data accuracy and reliability were guaranteed. All procedures involving human subjects received the approval of the Biomedical Ethics Committee of Peking University in China (IRB00001052-13074, 11/2021). The data were systematically assessed for reliability and validity, response rate, degree of missing information in the sample, and the rate of internal logical errors for the main health indicators [23].

**Figure 1:**
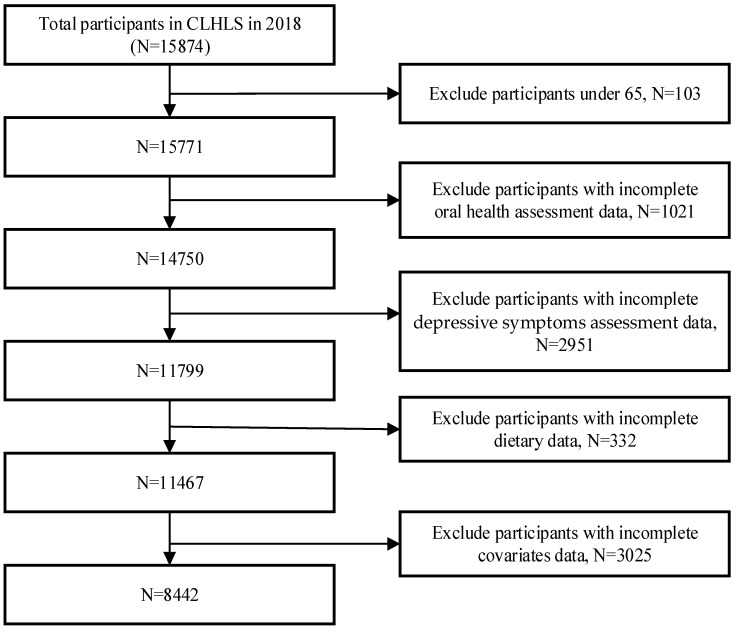
Flow chart for inclusion and exclusion of research participants.

In this study, participants who were tracked in 2018 were included. Finally, 8442 participants were included in this study in accordance with the inclusion and exclusion criteria in Figure 1.

### 2.2. Measures

#### 2.2.1. Assessment of Oral Health

Oral health included denture use and oral hygiene behavior. Denture use was obtained by asking the older adults if they wear dentures, and the answer of yes was assigned a value of 1, while the answer of no was assigned a value of 0. The oral hygiene behavior indicator was defined as how often the older adult brushed their teeth every day. The frequency of toothbrushing was assigned as 0, 1, 2, 3, and 4 for “never brush”, “brush occasionally”, “once a day”, “twice a day”, and “three or more times a day”, respectively.

#### 2.2.2. Assessment of Dietary Diversity

The DDS is used to assess the diversity of foods consumed by older adults. The DDS is calculated by the frequency of intake of 13 foods from the CLHLS questionnaire. These 13 foods include fresh fruits, vegetables, meat, fish, eggs, soy products, pickles, sugar, garlic, dairy products, nuts, and tea. The frequency of fresh fruit and vegetable intake included daily or almost daily, often, sometimes, and rarely or never. A score of 1 was given if the respondent’s intake frequency was daily or often, and a score of 0 was given if the respondent answered sometimes or almost never. The frequency of intake for the remaining 11 foods included five choices: almost every day, at least once a week, at least once a month, sometimes, rarely or never. Respondents received a score of 1 if they answered daily or weekly, and 0 if they answered monthly or sometimes or almost never [24]. The final DDS for each respondent was obtained by summing all food scores, ranging from 0 to 13. The higher the score, the more diverse the food intake. We defined a score less than 7 as low DDS and a score greater than or equal to 7 as high DDS [25].

#### 2.2.3. Assessment of Depressive Symptoms

In this study, the level of depressive symptoms in older adults, as assessed by the 10-item Center for Epidemiologic Studies Depression Scale (CESD-10) of the CLHLS, was used as the dependent variable. The Cronbach’s alpha for the CESD-10 was 0.84, showing sufficient reliability [26]. The scale includes seven questions representing negative emotions, such as “Do you feel sad or depressed” and “Do you feel nervous or scared”, which were positively scored by answering “never”, “rarely”, “sometimes”, “often”, or “always”. A score of 0 to 3 was assigned. The other three questions, such as “Do you feel as happy as you did when you were young”, representing positive emotions, were scored inversely. A total of 10 questions were scored in the range of 0–30, and a total score of ≥10 was defined as the presence of a depressive state [27], with a higher total score implying a higher level of depressive symptoms [28].

#### 2.2.4. Assessment of Covariates Variables

Based on previous investigations of factors influencing depressive symptoms [29,30], this study controlled for socio-demographic characteristics, socio-economic status, behavioral lifestyle, and health-status-related variables as covariates. The sociodemographic factors included sex, age, marital status, and residential status; the socioeconomic status included education and self-rated economic status; the behavioral lifestyle factors included smoking, alcohol consumption, and physical exercise status; and the health status factors included impaired activities of daily living (ADL) and serious illness.

### 2.3. Statistical Analysis

STROBE criteria were strictly followed in the statistical analysis (Table S1). Excel 2019 was used for data entry, and SPSS 24.0 was used for statistical analysis. The *t*-test, analysis of variance (ANOVA), and Kruskal–Wallis H-test were used to examine intergroup variability. Spearman correlation analysis was used to determine correlations between oral health assessment indicators, DD, and depressive symptoms. Mediation effect models were established by stepwise multiple linear regression analysis with denture use and toothbrushing frequency as independent variables, respectively, DDS as a mediator variable, depressive symptom score as a dependent variable, and statistically significant variables in the univariate analysis as control variables. Model 1 tested the relationship between the independent and dependent variables, Model 2 tested the relationship between the independent and mediating variables, and Model 3 tested the mediating role of DD. The PROCESS macro developed by Hayes was utilized to test for mediating effects, and the bias-corrected percentile Bootstrap (5000 replicated samples) was used to calculate 95% confidence intervals and indirect effects between variables. If the confidence interval did not contain 0, the mediating effect was shown to be statistically significant. *p* < 0.05 (two-sided) was considered statistically significant.

## 3. Results

### 3.1. Descriptive Analysis

A total of 8442 elderly samples were included in this study, of which 3878 (45.9%) were males and 4564 (54.1%) were females, with a mean age of the sample of 83.1 years, (SD, 11.2 years). The place of residence was mainly rural (74.0%). Dentures were present in the mouth of 3443 (40.8%) older adults, and the frequency of toothbrushing was the most frequently once a day in terms of oral care. The positive detection of depressive symptoms in the older adults was found in 3729 (44.1%). The depressive symptoms rate was 47.0% among older adults who did not use dentures and 52.7% among those who brushed their teeth occasionally. The differences in depressive symptoms by sex, age, urban-rural distribution, marital status, living arrangement, education level, self-rated economic status, smoking, alcohol consumption, exercise status, ability to perform daily activities, serious illness, and oral health were statistically significant (*p* < 0.001), as shown in Table 1.

### 3.2. Correlation Analyses of Denture Use, Toothbrushing Frequency, DD, and Depressive Symptoms

According to the results of Spearman’s correlation analysis (Table 2), denture use in the older adults was significantly and positively correlated with toothbrushing frequency (ρ = 0.251, *p* < 0.01) and DD (ρ = 0.113, *p* < 0.01), and significantly and negatively correlated with depressive symptoms (ρ = −0.077, *p* < 0.01); toothbrushing frequency and DD were significantly and positively (ρ = 0.268, *p* < 0.01) and significantly negatively correlated with depressive symptoms (ρ = −0.115, *p* < 0.01); there was a significant negative correlation between the DDS and depressive symptoms (ρ = 0.186, *p* < 0.01).

### 3.3. Mediating Role of DD in the Association between Oral Health and Depressive Symptoms

The correlation analysis showed that oral health status, DD, and depressive symptoms met the test conditions for mediating effects. After controlling for demographic characteristics and behavioral lifestyle variables, denture use had an effect on both depressive symptoms and DD (β = −0.341, 0.307. *p* < 0.001), and after introducing the DDS variable, both denture status and DDS had an effect on depressive symptoms (β = −0.294, −0.153. *p* < 0.001). The β for denture use decreased from −0.341 to −0.294, indicating that DD partially mediated the effect of denture use and depressive symptoms in older adults, as shown in Table 3.

**Figure 2:**
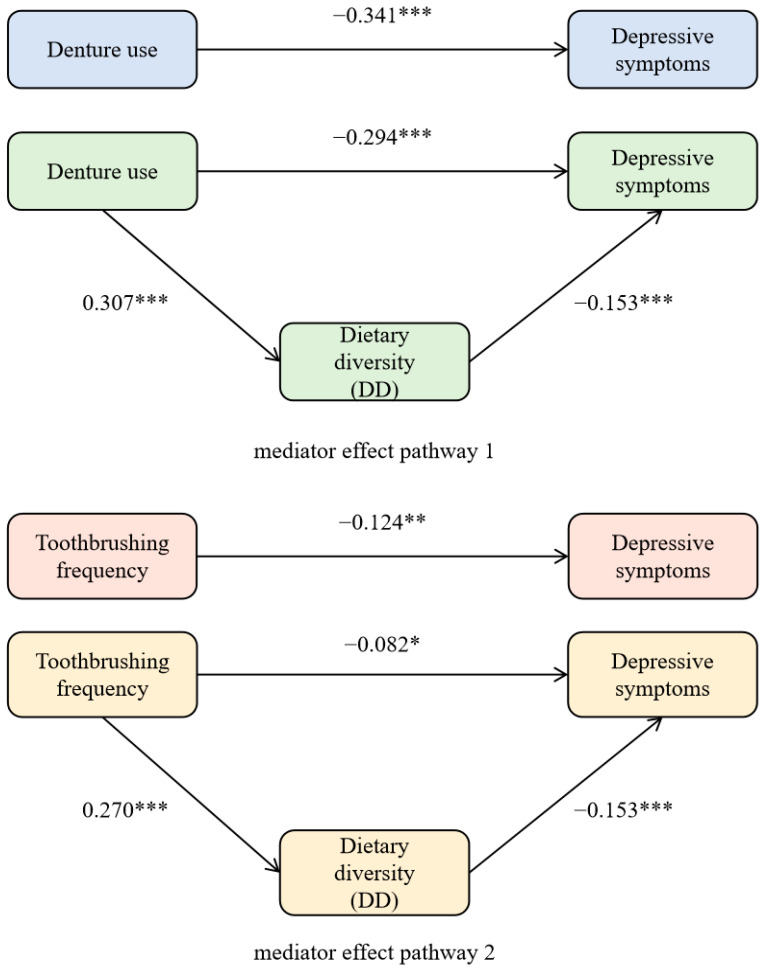
Intermediary effect roadmap. *** *p* < 0.001, ** *p* < 0.01, * *p* < 0.05.

Similarly, the β of frequency of toothbrushing on depressive symptoms was −0.124, and the β was −0.082 after adding DD to the regression equation; the partial regression coefficient decreased but was still significant, indicating that there was a mediating effect of DD in the prediction of the frequency of toothbrushing on depressive symptoms, see Table 4. The direct effect value was −0.294, and the total effect value for frequency of toothbrushing on depressive symptoms was −0.124 with a direct effect value of −0.082, suggesting a partial mediating effect of DD between denture condition and the frequency of toothbrushing and depressive symptoms, as shown in Figure 2.

A hypothesis test was conducted to test the mediating effect between DD in oral health status and depressive symptoms scores. As seen in Table 5, the 95% CI for the mediating effect of denture use via DD to depressive symptoms was [−0.068, −0.028]. The 95% CI for the mediating effect of toothbrushing frequency via DD to depressive symptoms is shown in Table 5. The 95% CI of the above two mediation pathways exclude 0, indicating that both mediation effects were significant. The mediating effects of DD between denture use and toothbrushing frequency and depressive symptoms accounted for 13.77% and 33.39%, respectively.

## 4. Discussion

The existing studies on oral health and depressive symptoms have mainly focused on their direct effects, and the internal mediating mechanisms have not been studied in depth [31]. The study is an extension of previous studies to explore the mechanisms of oral health on depressive symptoms in Chinese older adults through the introduction of DD. Moreover, in order to minimize the effect of recall bias, the denture use and average daily frequency of toothbrushing were chosen as oral health outcomes in this study, rather than the number of remaining teeth or tooth pain.

The results of this study showed that denture use among older adults negatively predict depressive symptoms, as validated by H1. Oral disease can affect overall health and is an integral part of overall health [32]. Denture use, on the other hand, can be physically and psychologically supportive for older adults. Tooth loss and even edentulousness problems are more prevalent in older adults. In contrast, the denture utilization rate among the older adults in this study was only 40.8%. Indeed, people need at least twenty functional teeth to chew food properly [33]. The absence of teeth effectively affects normal oral functions such as chewing and swallowing in the older adults, and the most common systemic diseases reported as a result include malnutrition, hypertension, and diabetes [34]. Also, the decline in the ability of older adults to chew and speak, as well as concerns about the exposure of unsightly dentition, can exacerbate their own social withdrawal and isolation [35], which can be detrimental to social participation and emotional communication, leading to lower self-esteem [11]. These factors are detrimental to the maintenance of a healthy lifestyle and promote the emergence of pain and stress, which can seriously affect their quality of life and thus increase the likelihood of developing depressive symptoms [36]. In contrast, the use of prostheses can have a remedial effect on oral function and body image in the older adults. Compared to edentulous patients, older adults with implant-retained overdentures (IRO) can improve maximum biting force and masticatory function, as well as promote an increase in occlusal muscle thickness [37]. The longer the duration of denture use, the better the masticatory performance [17]. Additionally, a more complete dentition contributes to increase the confidence in social communication and improved physical activity levels in older adults [35]. However, it has also been noted that wearing dentures may instead lead to poor oral health and even depressive symptoms [38]. This may be due to the high proportion of conventional dentures used by the older adults investigated, who are prone to shifting dentures that do not fit properly [39]. Once older adults experience discomfort such as oral pain, it can instead have a negative effect on their mental health.

The results of the mediating effect suggest that denture use can directly affect depressive symptoms in older adults and also indirectly through DD, as verified by H3. Previous studies have found that dentition, mastication, and nutrition are closely intertwined [33]. The weakening of chewing and swallowing ability by tooth loss may unconsciously change the food intake preferences of older adults by reducing the type and amount of food they consume. For example, they may prefer soft and easy-to-chew food [40] and may avoid fiber-rich food such as fruits, vegetables, and nuts [25,40,41]. The reduction in food variety and the high-fat, low-fiber nature of food intake has a direct effect on the physical and mental health of older adults. When investigating the Mini-Nutritional Assessment (MNA), they found that denture use increased the MNA score in older adults, with a higher proportion of diverse food intake and a higher rate of good nutrition than in edentulous patients [42]. And the diversity of food intake has a positive impact on depressive symptoms [22]. According to Maslow’s hierarchy, eating is the most basic human need. Food is associated with emotion and cultural identity [43]. Some studies have shown that omnivores possess better emotions than mono-dieters [44]. In addition, an increase in DDS is associated with a higher proportion of individuals consuming food groups, which contain high levels of B vitamins, and several epidemiological studies have reported a protective effect of these vitamins against depressive symptoms [45,46]. A varied diet may also prevent chronic diseases such as cancer [47], improve health status [48], and reduce the risk of death [49]. It effectively enhances the quality of life of older adults.

Spearman’s correlation analysis showed that toothbrushing frequency also negatively predicted depressive symptoms. When the mediating variable of DD was added, the direct effect of toothbrushing frequency on depressive symptoms was significantly reduced in older adults. This suggests that toothbrushing frequency not only significantly predicts depressive symptom levels, but also indirectly affects depressive symptom levels through DD. The results support H2 and H4. Regular toothbrushing as one of the indicators may reduce the likelihood of depressive symptoms in older adults. This is consistent with Oluwatoyin‘s findings [50]. Oral care patterns have an impact on poor oral health as well, with unhealthy oral care habits increasing the chances of caries [51,52]. Decreased brushing frequency worsens mental health and depressive symptoms. Poor oral hygiene due to reduced or irregular brushing is a predisposing factor for oral ulcers, necrotizing periodontal disease, and tooth loss [50]. The bad breath caused by it should not be ignored, which not only negatively affects the subjective perception of oral health, but also discourages the maintenance of interpersonal relationships and the development of interpersonal interactions [53]. Regular toothbrushing is a good hygiene practice that removes harmful bacteria from the oral cavity. This helps to maintain good oral health, increases the likelihood that older adults will consume a diverse range of foods, then reduces the risk of depressive symptoms. Furthermore, in another study exploring the association between oral health and mental health, the results found that depressive symptoms tended to be predisposed to higher rates of poor nutrition and poor oral hygiene [31]. Those older adults who experience depressive symptoms are more likely to have impaired health. They have a reduced ability to perform the activities of daily living and are more susceptible to chronic diseases. These can lead to poor oral behavior and a lower utilization of dental services [54].

Because the results support qualified appropriate denture use and brushing behaviors, as well as DD, there is a need to develop intervention strategies and measures to promote healthy aging. For example, policymakers should advocate for the inclusion of oral health in overall health strategies, encourage the inclusion of dental treatment costs in basic health insurance, and ensure equitable access to oral care services for older persons. Regular oral health clinics and dietary and nutritional education campaigns for older persons should be conducted in communities and towns, which not only provide timely attention to their oral and dietary problems, but also increase their social participation and prevent the onset and development of depressive symptoms. In fact, surveys show that approximately 75% of edentulous patients are recommended for complete prosthodontic treatment, which must include psychological counseling assessment and dietary guidance [55].

There are some limitations of this study. First, due to the limitations of the questionnaire available in the CLHLS database, standardized outcomes for dental exams, such as the “decayed, missing, and filled teeth” (DMFT) index or the brief oral health status examination (BOHSE) were not used as tools. Second, the type and frequency of food intake in this study were self-reported by older adults, so the results may be subject to recall bias. Third, due to the nature of cross-sectional studies, causality could not be accurately determined, nor can bidirectional associations be excluded. Finally, this study focused on the whole older adult population; the lack of subgroup analysis with different genders may affect the interpretation and generalization of the findings. Future investigations could recruit nutritionists and clinicians to participate in the survey, using objective measurement tools to improve the comprehensiveness and reliability of dietary, oral, and mental health assessments. After CLHLS updates the data, a longitudinal study to reveal the impact of time on the relationship between oral health, DD, and depressive symptoms may provide more in-depth research insights. In addition, considering subgroup analysis of sample populations with different cultural backgrounds or demographic characteristics, it is helpful to discover the causes behind the phenomenon and provide more powerful support for intervention decision-making.

## 5. Conclusions

Our findings suggest that denture use and toothbrushing frequency not only directly affect dietary diversity, but also negatively predict the emergence of depressive symptoms in Chinese older adults. To avoid the onset and progression of depressive symptoms, and ensure the diversity of the diet, policymakers or clinicians need to take steps to promote the preservation of natural teeth or dentures and oral hygiene among older adults and strengthen the partnership between psychiatric clinicians, dentists, and dietitians. While dentists are treating oral diseases, they assess the dietary and psychological conditions of older adults and provide feedback to dietitians and psychiatrists, and the three parties work together to develop dietary improvement and psychological intervention programs for those at high risk. Their dental health and DD are fully restored through health services to ensure overall health and well-being. In addition, future studies should focus on longitudinal evaluation of the effectiveness of dental disease and DD interventions in preventing or delaying the development of depressive symptoms, and explore their applicability in populations with different cultural backgrounds and gender groups.

## Figures and Tables

**Table 1:** Comparison of depressive symptoms scores of older adults with different basic conditions.

Variables	All (N = 8442)	Depressive Symptoms (N = 3729)	t/F/H Value	*p*-Value
Sex			−9.546	<0.001
Male	3878 (45.9%)	1532 (39.5%)		
Female	4564 (54.1%)	2197 (48.1%)		
Age			30.488	<0.001
65–74	2298 (27.2%)	853 (37.1%)		
75–84	2476 (29.3%)	1095 (44.2%)		
85–94	2082 (24.7%)	977 (46.9%)		
≥95	1586 (18.8%)	804 (50.6%)		
Residence			−5.724	<0.001
Urban areas	2194 (26.0%)	832 (37.9%)		
Rural areas	6248 (74.0%)	2897 (46.3%)		
Marital Status			−11.341	<0.001
With spouse	4015 (47.6%)	1535 (38.25%)		
No spouse	4427 (52.4%)	2194 (49.5%)		
Living arrangement			42.277	<0.001
Living with family	6761 (80.1%)	2583 (42.1%)		
Living alone	1396 (16.5%)	721 (51.6%)		
Nursing home	285 (3.4%)	155 (54.3%)		
Education			103.360	<0.001
No education experience	3654 (43.3%)	1882 (51.5%)		
Primary school	2931 (34.7%)	1191 (40.6%)		
Junior high school and above	1857 (22.0%)	656 (35.3%)		
Self-assessment of economic status			207.325	<0.001
Poorer	811 (9.6%)	537 (66.2%)		
General	5918 (70.1%)	2662 (44.9%)		
wealthier	1713 (20.3%)	530 (30.9%)		
Smoking			31.697	<0.001
No smoking	5775 (68.4%)	2713 (46.9%)		
Past smoking	1316 (15.6%)	480 (36.4%)		
Now smoking	1351 (16.0%)	536 (39.6%)		
Drinking			86.592	<0.001
No drinking	6129 (72.6%)	2858 (46.6%)		
Past drinking	1020 (12.1%)	417 (40.8%)		
Drinking now	1293 (15.3%)	454 (39.1%)		
Exercise			−16.117	<0.001
No	5432 (64.3%)	2699 (49.6%)		
Yes	3010 (35.7%)	1030 (34.2%)		
Serious illness			−7.160	<0.001
No	6353 (75.3%)	2707 (42.6%)		
Yes	2089 (24.7%)	1022 (48.9%)		
ADL			−11.114	<0.001
No difficulty	6916 (81.9%)	2911 (42.0%)		
Difficulty	1526 (18.1%)	818 (53.6%)		
Denture use			−6.893	<0.001
Yes	3443 (40.8%)	1376 (39.9%)		
No	4999 (59.2%)	2353 (47.0%)		
Toothbrushing frequency			127.890	<0.001
Hardly	1364 (16.2%)	712 (52.1%)		
Occasional	991 (11.7%)	523 (52.7%)		
Once a day	3537 (41.9%)	1493 (42.2%)		
Twice a day	2033 (24.1%)	831 (40.8%)		
Three or more times a day	517 (6.1%)	170 (32.8%)		

The t-value is the statistic of the *t*-test; the F-value is the statistic of the ANOVA; the H-value is the statistic of the Kruskal–Wallis H-test.

**Table 2:** Correlation analysis between denture use, frequency of toothbrushing and DD and depressive symptoms.

Variables	Denture Use	Toothbrushing Frequency	DD	Depressive Symptoms
Denture use	1.000			
Toothbrushing frequency	0.251 **	1.000		
DD	0.113 **	0.268 **	1.000	
Depressive symptoms	−0.077 **	−0.115 **	−0.186 **	1.000

** *p* < 0.01.

**Table 3:** Mediated model test of DD between denture use and depressive symptoms.

Pathway 1	Model 1		Model 2		Model 3	
Dependent Variable	Depressive Symptoms		DD		Depressive Symptoms	
Indicators	β	t	β	t	β	t
Denture use	−0.341	−4.094 ***	0.307	5.785 ***	−0.294	−3.540 ***
DD					−0.153	−8.989 ***
Sex	0.085	0.818	0.037	0.562	0.091	0.877
Age	0.027	0.534	−0.001	−0.034	0.027	0.533
Residence	0.081	0.780	−1.613	−24.384 ***	−0.166	−1.550
Marital status	0.206	1.937 *	−0.112	−1.657 *	0.189	1.783 *
Living arrangement	0.593	6.752 ***	−0.169	−3.022 ***	0.567	6.485 ***
Education	−0.202	−3.049 ***	0.518	12.256 ***	−0.123	−1.846 *
Economic status	1.486	19.040 ***	−0.735	−14.786 ***	1.373	17.457 ***
Smoking	−0.170	−2.622 ***	0.052	1.252	−0.162	−2.512 **
Drinking	−0.257	−4.195 ***	0.225	5.768 ***	−0.223	−3.643 ***
Exercise	−0.905	−10.064 ***	0.430	7.499 ***	−0.840	−9.347 ***
Serious illness	0.636	6.728 ***	−0.130	−2.156 **	0.616	6.547 ***
ADL	0.745	6.314 ***	0.181	2.409 **	0.773	6.577 ***
R-side	0.111		0.209		0.119	
F	80.865 ***		171.187 ***		81.571 ***	

*** *p* < 0.001, ** *p* < 0.01, * *p* < 0.05.

**Table 4:** Mediated model test of DD between toothbrushing frequency and depressive symptoms.

Pathway 2	Model 1		Model 2		Model 3	
Dependent Variable	Depressive Symptoms		DD		Depressive Symptoms	
Indicators	β	t	β	t	β	t
Toothbrushing frequency	−0.124	−3.041 **	0.270	10.474 ***	−0.082	−2.022 *
DD					−0.153	−8.937 ***
Sex	0.105	0.999	−0.014	−0.217	0.103	0.983
Age	0.010	0.197	0.036	1.115	0.015	0.307
Residence	0.029	0.275	−1.474	−21.844 ***	−0.196	−1.800 *
Marital status	0.208	1.954 *	−0.099	−1.470	0.193	1.820 *
Living arrangement	0.594	6.767 ***	−0.184	−3.302 ***	0.566	6.473 ***
Education	−0.178	−2.649 ***	0.458	10.764 ***	−0.108	−1.603
Economic status	1.492	19.115 ***	−0.717	−14.490 ***	1.383	17.576 ***
Smoking	−0.178	−2.741 ***	0.064	1.556	−0.168	−2.602 ***
Drinking	−0.253	−4.128 ***	0.220	5.647 ***	−0.220	−3.591 ***
Exercise	0.903	10.012 ***	−0.395	−6.895 ***	0.843	9.361 ***
Serious illness	0.627	6.635 ***	−0.133	−2.210 **	0.607	6.449 ***
ADL	0.713	6.024 ***	0.244	3.250 ***	0.751	6.364 ***
R-side	0.110		0.216		0.119	
F	80.865 ***		178.571 ***		80.888 ***	

*** *p* < 0.001, ** *p* < 0.01, * *p* < 0.05.

**Table 5:** Bootstrap mediated effect test of DD between oral health, depressive symptoms in older adults.

Category	Effect Value	Bootstrap SE	LLCI	ULCI	Effect Ratio
Denture use → total effect of depressive symptoms	−0.341	0.083	−0.505	−0.178	
Denture use → DD → depressive symptoms	−0.047	0.010	−0.068	−0.028	13.77%
Toothbrushing frequency → total effect of depressive symptoms	−0.124	0.041	−0.203	−0.044	
Toothbrushing frequency → DD → Depressive symptoms	−0.041	0.006	−0.054	−0.030	33.39%

## Data Availability

The original data presented in the study are openly available in Peking University Open Research Data at https://opendata.pku.edu.cn/dataverse/CHADS (accessed on 8 April 2024).

## Supplementary Materials

The following supporting information can be downloaded at: https://www.mdpi.com/article/10.3390/nu16081231/s1, Table S1: STROBE Statement—Checklist of items that should be included in reports of cross-sectional studies.
