# Supplementary material for: Association between Oral Health and Depressive Symptoms in Chinese Older Adults: The Mediating Role of Dietary Diversity
Source: Nutrients. 2024 Apr 21;16(8):1231. doi: 10.3390/nu16081231 (PMC11054946; doi:10.3390/nu16081231)
Supplement: Supplementary file 1 [file nutrients-16-01231-s001.zip › nutrients-2943299-supplementary.pdf]

Table S1. STROBE Statement—Checklist of items that should be included in reports of ***cross-sectional studies***.

	Item No	Recommendation
**Title and abstract**	1	(*a*) Indicate the study's design with a commonly used term in the title or the abstract<br>Line 13
		(*b*) Provide in the abstract an informative and balanced summary of what was done and what was found<br>Lines 9-24
**Introduction**		
Background/rationale	2	Explain the scientific background and rationale for the investigation being reported<br>Lines 28-70
Objectives	3	State specific objectives, including any prespecified hypotheses<br>Lines 71-79
**Methods**		
Study design	4	Present key elements of study design early in the paper<br>Lines 82-83
Setting	5	Describe the setting, locations, and relevant dates, including periods of recruitment, exposure, follow-up, and data collection<br>Lines 83-94
Participants	6	(*a*) Give the eligibility criteria, and the sources and methods of selection of participants<br>Lines 95-99 and Figure 1
Variables	7	Clearly define all outcomes, exposures, predictors, potential confounders, and effect modifiers. Give diagnostic criteria, if applicable<br>Lines 101-143
Data sources/ measurement	8*	For each variable of interest, give sources of data and details of methods of assessment (measurement). Describe comparability of assessment methods if there is more than one group<br>Lines 101-143
Bias	9	Describe any efforts to address potential sources of bias<br>Lines 88-90 and 230-232
Study size	10	Explain how the study size was arrived at<br>Lines 95-97
Quantitative variables	11	Explain how quantitative variables were handled in the analyses. If applicable, describe which groupings were chosen and why<br>Lines 120-122 and 132-134
Statistical methods	12	(*a*) Describe all statistical methods, including those used to control for confounding<br>Lines 145-160
		(*b*) Describe any methods used to examine subgroups and interactions<br>Not Applicable
		(*c*) Explain how missing data were addressed<br>Figure 1
		(*d*) If applicable, describe analytical methods taking account of sampling strategy<br>Not Applicable
		(*e*) Describe any sensitivity analyses<br>Not Applicable

**Results**		
Participants	13*	(a) Report numbers of individuals at each stage of study—eg numbers potentially eligible, examined for eligibility, confirmed eligible, included in the study, completing follow-up, and analysed<br>Figure 1
		(b) Give reasons for non-participation at each stage<br>Not Applicable
		(c) Consider use of a flow diagram<br>Figure 1
Descriptive data	14*	(a) Give characteristics of study participants (eg demographic, clinical, social) and information on exposures and potential confounders<br>Lines 163-174 and Table 1
		(b) Indicate number of participants with missing data for each variable of interest<br>Figure 1
Outcome data	15*	Report numbers of outcome events or summary measures<br>Table 1
Main results	16	(*a*) Give unadjusted estimates and, if applicable, confounder-adjusted estimates and their precision (eg, 95% confidence interval). Make clear which confounders were adjusted for and why they were included<br>Table 1
		(*b*) Report category boundaries when continuous variables were categorized<br>Table 1
		(*c*) If relevant, consider translating estimates of relative risk into absolute risk for a meaningful time period<br>Not Applicable
Other analyses	17	Report other analyses done—eg analyses of subgroups and interactions, and sensitivity analyses<br>Table 2, Table 3, Table 4 and Table 5
**Discussion**		
Key results	18	Summarise key results with reference to study objectives<br>Lines 233-234, 261-262 and 282-287
Limitations	19	Discuss limitations of the study, taking into account sources of potential bias or imprecision. Discuss both direction and magnitude of any potential bias<br>Lines 319-327
Interpretation	20	Give a cautious overall interpretation of results considering objectives, limitations, multiplicity of analyses, results from similar studies, and other relevant evidence<br>Lines 337-351
Generalisability	21	Discuss the generalisability (external validity) of the study results<br>Lines 226-232
**Other information**		
Funding	22	Give the source of funding and the role of the funders for the present study and, if applicable, for the original study on which the present article is based<br>Lines 358-359

*Give information separately for exposed and unexposed groups.
